# Third-Generation Antipsychotics as Augmentation in Treatment-Resistant Obsessive–Compulsive Disorder: A Narrative Review of Efficacy and Tolerability

**DOI:** 10.3390/biomedicines14010179

**Published:** 2026-01-14

**Authors:** Gianluca Rosso, Stefano Peracchia, Nicola Rizzo Pesci, Gabriele Di Salvo, Giuseppe Maina

**Affiliations:** 1 Department of Neurosciences ‘Rita Levi Montalcini’, University of Torino, 10126 Turin, Italy; gianluca.rosso@unito.it (G.R.); stefano.peracchia@unito.it (S.P.); gabriele.disalvo@unito.it (G.D.S.); giuseppe.maina@unito.it (G.M.); 2 Psychiatric Unit, San Luigi Gonzaga University Hospital, 10043 Orbassano, Italy

**Keywords:** obsessive–compulsive disorder, antipsychotic augmentation, treatment resistance

## Abstract

**Background/Objectives**: Obsessive–compulsive disorder (OCD) is a chronic psychiatric illness with intrusive obsessions and compulsive behaviors severely impacting daily functioning and quality of life. The purpose of this narrative review is to present an updated summary of available evidence on third-generation antipsychotics (TGAs) as augmentation strategies for SRI-refractory OCD. **Methods**: The literature was reviewed using the PubMed database to recognize studies on the use of TGAs in treatment-resistant OCD. Only articles in the English language and on human participants were included. **Results**: We included nine reports in our review. More numerous (five reports) and higher evidence-level reports were retrieved for aripiprazole, which consistently shows high response rates compared to placebo and other antipsychotics. Two cohort studies were included on brexpiprazole, with no active or placebo comparator. These showed varying but high response rates. One cohort study reported a response rate of 61.5% to cariprazine. Only one paper reported on the efficacy of lumateperone in OCD. This was a single-case report on an adolescent patient with refractory OCD responding to lumateperone monotherapy. **Conclusions**: The current state of evidence supports the clinical utility of TGAs, particularly aripiprazole, in augmenting SRI treatment in patients with refractory OCD. Evidence regarding cariprazine and lumateperone is scarce, but still contributes to the discussion on the use of TGAs in OCD.

## 1. Introduction

Obsessive–compulsive disorder (OCD) is a pervasive and sometimes disabling mental disorder defined by the experience of recurrent intrusive thoughts (obsessions) and repeated behaviors or mental actions (compulsions) aimed at alleviating anxiety or averting feared harmful outcomes [1]. The disorder is present in about 2–3% of the population, typically developing in the teenage or young adult stages of life, and is characterized by significant functional disability and impaired quality of life [2,3].

First-line therapies are generally considered to be high-dose serotonin reuptake inhibitors (SRIs) and cognitive behavioral therapy with exposure and response prevention (CBT-ERP). These strategies are effective in a significant percentage of patients [4]. Response to treatment is conventionally defined as a decline of at least 25% in the Yale–Brown Obsessive–Compulsive Scale (Y-BOCS) total score. SRI-refractory OCD is defined as failure to achieve clinically significant response following at least 12 weeks of adequate SRI treatment [5]. It is estimated that 40–60% of patients are partial or nonresponders to SRI treatment even at adequate dosages and treatment duration [2,5]. This clinical issue has stoked interest in pharmacologic augmentation strategies to enhance treatment efficacy.

Augmentation with antipsychotic medication has yielded a strong evidence, specifically related to the use of second-generation medications like risperidone [6,7]. Nonetheless, their use is often limited by side effects like metabolic problems and extrapyramidal symptoms [8]. More recently, third-generation antipsychotics (TGAs), namely aripiprazole, brexpiprazole, cariprazine, and lumateperone, have emerged as reliable substitutes based on their unique receptor activity profile, holding promise to offer efficacy as well as greater tolerability [9].

TGAs represent a recent class of antipsychotic agents, primarily encompassing aripiprazole, brexpiprazole, cariprazine, and lumateperone. Unlike first-generation (typical) and second-generation (atypical) antipsychotics, which are primarily characterized by dopamine D_2_ receptor antagonism (without or with serotonin 5-HT_2_A antagonism, respectively), TGAs are defined by their dopamine receptor functional selectivity and partial agonism, offering a novel approach to modulating dopaminergic neurotransmission [10]. Their underlying property is dopamine D_2_/D_3_ partial agonism and, therefore, are able to act as dopamine system stabilizers.

Aripiprazole is a partial agonist at the D_2_ receptor and at the 5-HT_1_A receptor and an antagonist at 5-HT_2_A receptors. It modulates the dopaminergic tone and elevates serotonergic activity [11]. Its pharmacologically close analogue brexpiprazole is less intrinsically active at D_2_ receptors and has a greater antagonist effect on 5-HT_2_A and α_1_B/α_2_C adrenergic receptors, has less risk of akathisia, and seems to have higher tolerability [12].

Cariprazine has a higher affinity for D_3_ than D_2_ receptors and has preferential actions on mesolimbic reward and motivational systems, showing lower rates of nigrostriatal side effects compared to other antipsychotics [13].

Lumateperone has concurrent moderate D_2_ receptor partial agonism and a strong 5-HT_2_A antagonism and serotonin transporter blockade; it also indirectly augments NMDA receptor signaling through D1-dependent glutamatergic modulation [14].

TGAs’ partial agonism at D_2_ receptors prevents dopaminergic blockade in striatal circuits, and balanced serotonergic action enhances mood/anxiety stabilization and tolerability, having fewer dopaminergic side effects such as extrapyramidal symptoms (EPS) and hyperprolactinemia. Lower liability to metabolic and extrapyramidal side effects and potential pro-cognitive and anti-obsessional actions make them strong candidates for treatment of substance-induced psychosis [15] as well as treatment-resistant OCD augmentation strategies [16].

This narrative review will synthesize the evidence to date on the efficacy and tolerability of third-generation antipsychotics employed as augmentation agents in SRI-refractory OCD.

## 2. Materials and Methods

The literature was reviewed through a search on the PubMed database to retrieve studies produced from inception to May 2025. The search strategy used the following keywords: “obsessive–compulsive disorder,” “OCD,” “aripiprazole,” “brexpiprazole,” “cariprazine,” “lumateperone,” “third-generation antipsychotics,” “dopamine partial agonists,” and “augmentation.” Only articles in the English language and on adult human participants were included.

Included trials were randomized controlled trials, cohort studies, cross-sectional studies, descriptive reports, case series, and case reports reviewing the augmentation therapy by third-generation antipsychotics in subjects with OCD. When meta-analyses reported aggregate data from previous trials, the latter were not additionally discussed. The exclusion criteria were as follows: failure to report clinical outcomes on the severity of symptoms in OCD, antipsychotic therapy as a monotherapy, preclinical or animal experiments, and non-peer-reviewed publications.

In conformity with the existing clinical convention, response to treatment was defined as a decline by at least 25% in the Yale–Brown Obsessive–Compulsive Scale (Y-BOCS) total score at onset of the augmentation period. SRI-refractory OCD was defined as failure to achieve clinically significant response following at least 12 weeks of adequate SRI treatment [5].

## 3. Results

A total of nine reports were included in this review (Figure S1). Of these, five investigated the efficacy of aripiprazole, including one network meta-analysis, one randomized controlled trial, and three cohort studies. Two retrospective observational studies on brexpiprazole were retrieved. One cohort study reported on the effects of cariprazine and one case report discussed the efficacy of lumateperone in OCD. The latter was included despite being a case report of an adolescent patient, as it was the only available report on the use of lumateperone in OCD (Table 1).

Two observational cohort studies on the use of aripiprazole as augmentation in SRI-resistant OCD were conducted in 2011. In the study by Ak and colleagues [17], 23 SRI-resistant OCD patients were treated with aripiprazole and, at the 10-weeks endpoint, 30.4 had showed a clinical response. A similar design was adopted by Delle Chiaie and colleagues [18], who recorded a much larger response rate of 80%.

In 2015, Shoja and colleagues conducted an RCT, which is not included in subsequent meta-analyses. Forty-four participants were randomized 1:1 to either quetiapine or aripiprazole augmentation. Despite both groups showing a decrease in Y-BOCS scores, this was not significant for the aripiprazole group [19].

The 12-week prospective observational study by Di Salvo et al. evaluated the efficacy and tolerability of aripiprazole for obsessive–compulsive symptoms in euthymic bipolar patients with comorbid OCD. The mean reduction in the Y-BOCS score in completers was from 24.0 ± 4.1 to 17.1 ± 4.3 (*p* < 0.001). The response rate (≥35% reduction in Y-BOCS) was 41.8%, while an additional 18.2% showed a partial response. It is interesting to note that in this population, around 90% of patients experienced at least one adverse effect (most common: tremor, tension/internal restlessness, reduced sleep, akathisia), and 21.4% discontinued treatment due to AEs within the first 6 weeks [20].

The network meta-analysis by Maiti et al. in 2023 [21] analyzed 59 randomized trials to evaluate the efficacy of pharmacological augmentation strategies with SRI in resistant OCD. It included five studies on aripiprazole augmentation [22,23,24,25,26]. The results show that aripiprazole is associated with a significant reduction in the Y-BOCS score (MD −5.4; 95% CrI −9.1, −1.6), while risperidone has a more modest but still significant effect (MD −3.3; 95% CrI −6.4, −0.20). Interestingly, the meta-regression shows that the efficacy of antipsychotics is greater in SRI-refractory patients, regardless of the duration of augmentation therapy [21].

**Table 1:** List of included studies and their main characteristics.

Year	Authors	Study Design	Intervention	Comparator	Sample	N	Main Outcome
2011	Ak M et al. [17]	Cohort study	Aripiprazole	None	SRI-resistant OCD patients	23	30.4% of patients showed a response at 10 weeks
2011	Delle Chiaie R et al. [18]	Cohort study	Aripiprazole	None	Treatment-resistant OCD patients on SSRI or clomipramine	20	80% of the patients showed a full response to aripiprazole, and 10% showed a partial response at 12 weeks
2015	Shoja S S and Kaviani H [19]	RCT	Aripiprazole	Quetiapine	Female inpatients with fluvoxamine-resistant OCD	Aripiprazole: 22 quetiapine: 22	The aripiprazole group did not show a statistically significant improvement, while the quetiapine group did
2020	Di Salvo G et al. [20]	Cohort study	Aripiprazole	None	Euthymic BD patients with comorbid OCD	70	Y-BOCS reduction from 24.0 (+/−4.1) at baseline to 17.1 (+/−4.3) at 12 weeks
2023	Maiti R et al. [21]	Network Meta-analysis	Aripiprazole	Placebo, olanzapine, quetiapine, risperidone	OCD patients receiving augmentation to SSRIs or clomipramine	Aripiprazole: 96	−5.4 (−9.1, −1.6) reduction of Y-BOCS with aripiprazole compared to placebo; no significant difference compared to olanzapine, quetiapine, risperidone
2024	Martiadis V et al. [27]	Cohort study	Brexpiprazole	None	SRI-resistant OCD patients	34	50% of participants had a clinical response at 12 weeks
2024	Martiadis V et al. [28]	Cohort study	Cariprazine	None	SRI-resistant OCD patients	13	61.5% of participants showed a response at 12 weeks
2023	Naguy A et al. [29]	Case report	Lumateperone	None	Adolescent with treatment-resistant OCD	1	Lumateperone monotherapy led to remission in a treatment-resistant OCD patient
2025	Giacovelli L et al. [30]	Cohort study	Brexpiprazole	None	Treatment-resistant OCD patients on SRIs	10	70% of patients had a clinical response at 12 weeks

Despite its relatively recent introduction, brexpiprazole also shows potential in the treatment of OCD with characteristics of resistance to conventional treatments. In a 2024 retrospective observational study conducted in Italy by Martiadis et al. on 34 adult patients with treatment-resistant OCD, patients received brexpiprazole in addition to their current treatment for 12 weeks (initial dose 1 mg/day, increasable to 2–3 mg; the majority of participants remained at 1 mg/day). At the end of the study, 17 patients (50%) met the response criteria. Of these, 10 patients (~29% of the total sample) achieved a ≥35% reduction in symptoms. No serious adverse events were reported [27]. In another independent retrospective study conducted in Milan on 10 adult patients with SSRI-resistant OCD, brexpiprazole (starting dose 1 mg/day) was added for 12 weeks leading to significant clinical improvement after 3 months: 7 patients (70%) achieved at least a 25% reduction in Y-BOCS, including 5 patients (50%) with a robust response of ≥35% reduction. Only 2 patients experienced adverse effects [30].

New evidence regarding cariprazine and lumateperone also reignites the discussion on TGAs in OCD augmentation.

In their retrospective observational study of 13 patients with treatment-resistant obsessive–compulsive disorder (OCD), Martiadis et al. examined the effectiveness of augmenting ongoing serotonin reuptake inhibitor (SRI) therapy with low-dose cariprazine. Over a 12-week period, participants showed significant reductions in the Yale–Brown Obsessive–Compulsive Scale (Y-BOCS) total scores (*p* < 0.001), including both obsession and compulsion subscales, as well as significant improvement in the Clinical Global Impression-Severity scores (*p* < 0.001). At the endpoint, 61.5% of patients met response criteria (≥25% Y-BOCS improvement), a rate higher than typically reported for other antipsychotic augmentations. Cariprazine was generally well tolerated; adverse events were mild and did not lead to discontinuation, with the most common being a sense of inner tension [28]. A case report from Naguy et al. (2023) [29] was included despite being on an adolescent patient, because it was the only record retrieved on the use of lumateperone in OCD. It describes the case of a 15-year-old patient with severe and highly resistant OCD, unresponsive to multiple treatments (high-dose SSRIs, clomipramine, CBT, risperidone, aripiprazole, augmentation with lithium, and other strategies). After drug washout, lumateperone monotherapy was initiated at 42 mg/day, with rapid and marked improvement (a 61% of Y-BOCS score reduction) in the first 4 weeks and further stabilization in the mild range in the following months, with excellent tolerability, normalization of prolactin, and weight loss. The authors highlight the possible role of lumateperone in refractory cases thanks to its multimodal profile (5-HT2A antagonism, dopaminergic and glutamatergic modulation), although this is a single observation that requires systematic confirmation [29].

## 4. Discussion

Cumulative evidence from the reviewed literature suggests that third-generation antipsychotics appear to be promising augmentation therapies in treatment-resistant OCD. This is in accordance with the expected clinical signs of the pharmacological effects of third generation antipsychotics, and specifically based on their novel mechanisms of dopaminergic modulation. Aripiprazole demonstrated the firmest evidence for its efficacy, with variable response rates (up to 80% in some reports), with acceptable tolerability. The favorable effect on OCD symptoms, evidenced by the significant improvement in Y-BOCS scores, is well-documented in clinical trials, meta-analyses, and confirmed from the pharmacological benefits associated with its partial agonism at the level of the dopamine receptor, in addition to action on the serotonergic system [31,32,33].

Even if some evidence tends to show that risperidone could occasionally have better efficacy, the risk–benefit evaluation favors the use of aripiprazole in treatment-resistant forms, in view of its mild spectrum of side effects combined with a low tendency to induce metabolic complications [34,35].

Clinical evidence on brexpiprazole in treatment-resistant OCD is still limited compared to aripiprazole; however, the available data are promising and show a favorable efficacy profile, with good efficacy and response rates higher than 35% [30]. Side effects are generally mild and well tolerated (sedation, moderate weight gain). Brexpiprazole has a more complex receptor profile, with even more pronounced 5-HT1A agonism, which could translate into superior efficacy in subgroups of patients with depressive or anxiety comorbidities, although further randomized controlled trials are needed to consolidate the current evidence and determine the clinical utility of brexpiprazole in the management of treatment-resistant OCD [27,30].

Cariprazine is a unique candidate for augmentation in treatment-resistant OCD due to its partial agonism with affinity for D3 dopaminergic receptors, which are abundant in the limbic system, and partial agonism on 5-HT1A serotonergic receptors, in addition to 5-HT2A antagonism. This modulating combination acts effectively on the dysfunctional dopaminergic and serotonergic circuits in OCD, with a pharmacokinetic profile that includes active metabolites with a long half-life, ensuring stability and therapeutic continuity. These characteristics could make cariprazine suitable for modulating motivational and emotional aspects of treatment-resistant OCD with a potential advantage over other antipsychotics [36]. Similarly, there is initial evidence that lumateperone’s multimodal mechanism, which includes modulation of glutamatergic as well as dopaminergic and serotonergic pathways, may offer a therapeutic alternative for patients not responding to conventional strategies; however, current evidence derives from a single-case observation, and is far from strong and conclusive [29]. The evidence on lumateperone needs to be considered as a hint to a potential therapeutic role of the molecule, rather than as a base for its clinical application as of now. Lumateperone is pharmacologically distinct from other atypical antipsychotics and offers a particularly intriguing multitarget mechanism of action for the neurobiology of treatment-resistant OCD: it exerts its modulatory function by acting as a potent serotonergic 5-HT2A antagonist, with an affinity for the 5-HT2A receptor 60 times higher than that for the D2 dopaminergic receptor. In OCD, the hyperactivity of orbitofrontal circuits is often sustained by excessive excitatory tone. A 5-HT2A blockade would reduce downstream glutamate release, “cooling” the cortico-striatal hyperactivity responsible for obsessions, without heavily blocking dopamine nor causing emotional flattening. The drug is also a mild serotonin transporter (SERT) inhibitor, acting similarly to SSRIs. The action of lumateperone on the glutamatergic system is extremely sophisticated and is thought to mediate synaptic plasticity and long-term potentiation, neurobiological processes crucial for fear extinction and cognitive flexibility, which are often impaired in treatment-resistant OCD [37,38].

The favorable tolerability profile of TGA observed in the aforementioned literature highlights the potential benefits of TGAs when used in sensitive populations such as adolescents [29]. These observations are critical, as older augmentation agents such as risperidone, while efficacious, have been associated with a higher burden of adverse metabolic and extrapyramidal side effects, thus limiting their long-term use [5,31]. Third-generation antipsychotics (TGAs) such as aripiprazole, cariprazine, brexpiprazole, and lumateperone were developed specifically to improve the tolerability of earlier agents through partial dopamine agonism and more selective receptor profiles. Nevertheless, despite generally showing lower risks of metabolic adverse effects (weight gain, dyslipidemia, and glucose dysregulation) and reduced prolactin elevation compared with many SGAs (e.g., olanzapine, risperidone, quetiapine, and paliperidone), TGAs are commonly associated with akathisia and restlessness, particularly with aripiprazole and cariprazine [39]. Aripiprazole tends to have the lowest extrapyramidal symptom (EPS) profile apart from akathisia, while brexpiprazole may cause more weight gain among TGAs [40]. Lumateperone is notable for minimal weight gain and metabolic change, with somnolence being one of the more frequent side effects.

The present literature review is limited by its narrative nature. It was not systematic in terms of search and study quality assessment, as it was meant to provide an overview on an emerging subject. The scope of this perspective article is to explore a rising topic for which little evidence, largely from the lower levels of the pyramid of evidence, is available. We collected the available evidence and summarized it rapidly to foster new research on the topic; therefore, time and resources were not allocated to thorough and systematic appraisal of the evidence or risk of bias assessment Moreover, the conclusions drawn are hindered by the small number of studies, which are mostly observational. Significant gaps in current evidence are worth mentioning: given the absence of RCTs on newer antipsychotics for this indication, the current literature is primarily composed of observational studies, small-scale randomized trials, and case reports, all of which emphasize the need for more robust research to confirm and extend these findings. There is only one trial registered on clinicaltrials.gov investigating TGA augmentation in treatment-resistant patients, and it is specifically on aripiprazole (NCT04539951) [41]. Another trial including an aripiprazole arm (NCT02955654) was withdrawn [42]. Another RCT on treatment-resistant OCD is registered on the ISRCTN portal (ISRCTN66385119) [43]. This trial investigates the efficacy of a three-step treatment approach based on CBT, CBT + sertraline, and ultimately CBT + sertraline + aripiprazole. Multiple result papers on the CBT steps are available, but not on the results of aripiprazole augmentation [44].

Until further studies are available, clinicians must base their treatment decisions on a judicious evaluation of the limited data, individual patient characteristics, previous treatment responses, and the potential risk–benefit ratio of each augmentation strategy. Investigators are encouraged to conduct multicenter, randomized, double-blind studies to assess the efficacy and tolerability of these medications beyond aripiprazole, investigating the clinical utility of cariprazine, brexpiprazole, and potentially lumateperone in the context of OCD augmentation. Moreover, stratified analyses might prove valuable to identify patient characteristics that could predict a favorable response to TGA augmentation, such as the presence or absence of comorbid tic disorders, specific OCD symptom dimensions, and differences in gender and treatment history. Nevertheless, the discussed data on the use of third generation antipsychotics in OCD are encouraging, as they could pave the way to more efficacious and tolerable management strategies in SRI refractory patients.

## 5. Conclusions

Overall, this narrative review suggests that third-generation antipsychotics represent a promising and potentially better-tolerated augmentation strategy for SRI-refractory OCD. Aripiprazole shows the most robust evidence to date, while emerging data on brexpiprazole, cariprazine, and lumateperone are encouraging but preliminary. Well-designed randomized controlled trials are needed to clarify their comparative efficacy, safety, and optimal patient selection.

## Data Availability

No new data were created or analyzed in this study.

## Supplementary Materials

The following supporting information can be downloaded at: https://www.mdpi.com/article/10.3390/biomedicines14010179/s1, Figure S1: Flowchart of assessed and included reports.

## Abbreviations

The following abbreviations are used in this manuscript:

OCD	Obsessive–compulsive disorder
TGA	Third-generation antipsychotics
SRI	Serotonin reuptake inhibitors
SSRI	Selective serotonin reuptake inhibitors
CBT-ERP	Cognitive behavioral therapy with exposure and response prevention
Y-BOCS	Yale–Brown Obsessive–Compulsive Scale
