# Supplementary figures and images for: Third-Generation Antipsychotics as Augmentation in Treatment-Resistant Obsessive–Compulsive Disorder: A Narrative Review of Efficacy and Tolerability
Source: Biomedicines. 2026 Jan 14;14(1):179. doi: 10.3390/biomedicines14010179 (PMC12838634; doi:10.3390/biomedicines14010179)

**Figure S1.** Flowchart of assessed and included reports.

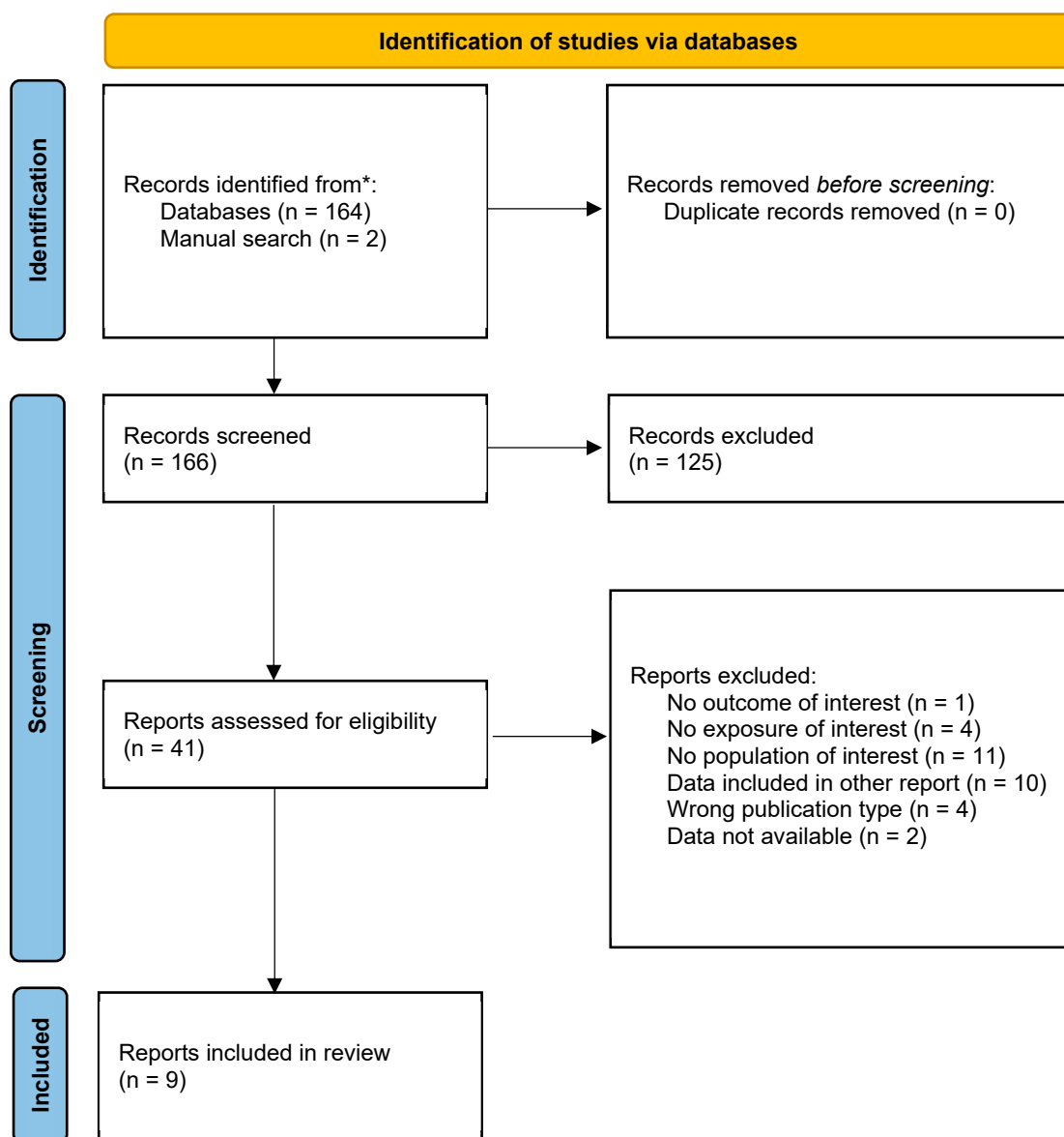

Supplement: Supplementary file 1 [file biomedicines-14-00179-s001.zip › biomedicines-4033847-supplementary.pdf]
